# Decreased temperature increases the expression of a disordered bacterial late embryogenesis abundant (LEA) protein that enhances natural transformation

**DOI:** 10.1080/21505594.2021.1918497

**Published:** 2021-05-03

**Authors:** Terhi Maula, Nelli Vahvelainen, Helena Tossavainen, Tuuli Koivunen, Marja T. Pöllänen, Anders Johansson, Perttu Permi, Riikka Ihalin

**Affiliations:** a Department of Life Technologies, University of Turku, Turku, Finland; b Department of Biological and Environmental Sciences, Nanoscience Center, University of Jyvaskyla, Jyvaskyla, Finland; c Institute of Dentistry, University of Turku, Turku, Finland; d Division of Molecular Periodontology, Department of Odontology, Umeå University, Umeå, Sweden; e Department of Chemistry, Nanoscience Center, University of Jyvaskyla, Jyvaskyla, Finland

**Keywords:** Cold shock protein, late embryogenesis abundant protein, *Aggregatibacter actinomycetemcomitans*, DNA transformation competence, NMR spectroscopy

## Abstract

Late embryogenesis abundant (LEA) proteins are important players in the management of responses to stressful conditions, such as drought, high salinity, and changes in temperature. Many LEA proteins do not have defined three-dimensional structures, so they are intrinsically disordered proteins (IDPs) and are often highly hydrophilic. Although LEA-like sequences have been identified in bacterial genomes, the functions of bacterial LEA proteins have been studied only recently. Sequence analysis of outer membrane interleukin receptor I (BilRI) from the oral pathogen *Aggregatibacter actinomycetemcomitans* indicated that it shared sequence similarity with group 3/3b/4 LEA proteins. Comprehensive nuclearcgq magnetic resonance (NMR) studies confirmed its IDP nature, and expression studies in *A. actinomycetemcomitans* harboring a red fluorescence reporter protein-encoding gene revealed that *bilRI* promoter expression was increased at decreased temperatures. The amino acid backbone of BilRI did not stimulate either the production of reactive oxygen species from human leukocytes or the production of interleukin-6 from human macrophages. Moreover, BilRI-specific IgG antibodies could not be detected in the sera of *A. actinomycetemcomitans* culture-positive periodontitis patients. Since the *bilRI* gene is located near genes involved in natural competence (i.e., genes associated with the uptake of extracellular (eDNA) and its incorporation into the genome), we also investigated the role of BilRI in these events. Compared to wild-type cells, the Δ*bilRI* mutants showed a lower transformation efficiency, which indicates either a direct or indirect role in natural competence. In conclusion, *A. actinomycetemcomitans* might express BilRI, especially outside the host, to survive under stressful conditions and improve its transmission potential.

## Introduction

Proteins belonging to the late embryogenesis abundant (LEA) family can be found in *Archaea, Bacteria* and *Eucarya* (predominantly in plants). Although these proteins have been intensively studied, the structural basis of their functions is poorly understood. LEA proteins exhibit critical functions linked to the ability to withstand stressful conditions such as cold temperatures and dehydration. The LEA protein family is divided into various subgroups based on sequence similarity. Known bacterial LEA proteins belong to groups LEA_2, LEA_4 and LEA_5 (following Pfam classification) [1,2]. Although their functions have not been extensively studied, these proteins play roles in resistance to abiotic stresses, including cold, freezing, desiccation and oxidation [3–9]. Many LEA proteins are intrinsically disordered and highly hydrophilic. The sequence analysis of prokaryotic LEA proteins has revealed that these sequences are not usually located in functional genomic islands but exhibit random locations in the genome and are distributed among bacteria via horizontal gene transfer (HGT) [2].

Periodontitis is a common bacterial biofilm infection that destroys tooth-supporting tissues, leading in the worst-case scenario to the loss of the affected tooth. Periodontitis is classified into different categories based on its pathophysiology, stage, and grade [10]. Although periodontitis-related subgingival biofilms comprise various bacterial species, the majority of which are gram negative, some species may play a more pronounced role than others in the development of dysbiosis, leading to inflammatory reactions and the destruction of host tissues and alveolar bone (for a review, see [11]). One of these periodontal pathogens, *Aggregatibacter actinomycetemcomitans*, is potentially aggressive and possesses many virulence factors, such as long bundled fimbriae, adhesion factors, cytolethal distending toxin (CDT), leukotoxin, lipopolysaccharide (LPS), and peptidoglycan-associated lipoprotein (PAL), which play roles in colonization and biofilm formation, induce inflammation and help the opportunistic pathogen to evade the host immune defense system [12–14]. Moreover, some strains of *A. actinomycetemcomitans* are naturally competent; that is, they can take up eDNA and incorporate it into their genome [15,16]. For natural transformation to be possible, the species must exhibit a functional Type IVa pilus machine and proteins involved in homologous recombination, comprising Com (A, B, C, D, E, EA, F, E1), Pil (A, B, C, D), Rec2, ComM, and UrpA proteins [15,17–19]. Naturally, competent bacteria can use eDNA as a nutrient or as a source of novel genes to increase their robustness and virulence. *A. actinomycetemcomitans* displays virulence potential not only in the oral cavity but also in other parts of the body and is associated with abscesses in the brain [20], endocarditis [21] and rheumatoid arthritis [22].

The composition of the outer membrane (OM) is critical for various functions of gram-negative bacteria; in potential pathogens in particular, OM components such as LPS, proteins and lipoproteins play important roles in interactions with host cells. The abovementioned molecules have traditionally been considered proinflammatory agents, especially when released in vesicles, and they may operate as virulence factors that hamper host defense [23]. For instance, LPS is a well-known proinflammatory agent that sequesters the neutrophil-attracting chemokine interleukin (IL)-8 [24]. Similarly, various OM proteins (OMPs) of gram-negative bacteria have the potential to bind host inflammation-related cytokines/chemokines [25–28]. In the *A. actinomycetemcomitans* OM, we previously identified a lipoprotein that we named bacterial interleukin receptor I (BilRI) since it interacted with IL-1β [29] and various other cytokines/chemokines [30]. However, the biological role of this intrinsically disordered OMP as a biologically significant chemokine binder has been challenged by the finding that abundant *A. actinomycetemcomitans* LPS binds IL-8 with a higher affinity than BilRI [24].

Intrinsically disordered proteins (IDPs) do not exhibit well-defined structures in solution, and they may adopt various 3-dimensional structures when interacting with different ligands [31]. Our earlier study showed that BilRI was disordered in solution, which was supported by the presence of characteristic repeated sequences devoid of bulky hydrophobic amino acids, as well as unconventionally high solubility in water [30]. Despite the increased dynamics of IDPs and the lack of stable 3-dimensional conformations in these proteins, it is possible to determine the positions of transient secondary structure elements and conformational ensembles using biophysical tools such as nuclear magnetic resonance (NMR) spectroscopy, small angle X-ray scattering (SAXS) and single-molecule Förster resonance energy transfer (smFRET) [32].

Information about the structural details of a protein may help define its role in the pathogen. Therefore, the main aim of this study was to shed light on the conformational properties of intrinsically disordered BilRI using solution-state NMR spectroscopy. Moreover, we studied whether BilRI, as an OMP facing the extracellular space, could be involved in cell–cell interactions via contact with host leukocytes and macrophages and whether BilRI is immunogenic in *A. actinomycetemcomitans-*positive periodontitis patients. In addition, we had no previous information about BilRI expression levels under different conditions. *A. actinomycetemcomitans* produces proteins that may bind various IgG molecules [33], and commercial antibodies against its proteins are unavailable. Therefore, protein expression studies based on detection with IgG antibodies could be problematic, especially when a low expression level is anticipated. Thus, we produced a mutant *A. actinomycetemcomitans* strain devoid of the *bilRI* gene that expressed the fluorescent reporter protein DsRed-monomer under the control of the genomic *bilRI* promoter. By using this mutant strain, we investigated the regulation of the *bilRI* gene. We also mapped the location of the *bilRI* gene in the genome of naturally competent *A. actinomycetemcomitans* D7S and investigated the possible role of BilRI in natural transformation.

## Materials and methods

### Ethics statement

Permission to collect and use blood samples of *A. actinomycetemcomitans*-positive periodontitis patients and healthy control subjects was obtained from the Ethics Committee of the Hospital District of Southwest Finland. Written informed consent was obtained from *A. actinomycetemcomitans*-positive adult periodontitis patients (21) and healthy controls (13) to collect venous blood samples.

## BilRI sequence analysis

Sequence similarity searches were performed with NCBI BLAST [34] using the amino acid sequence of *A. actinomycetemcomitans* strain D7S BilRI as a template. Multiple sequence alignments were performed using Clustal Omega [35] and edited using the BioEdit sequence alignment editor (Informer Technologies, Inc.).

## Production and purification of recombinant BilRI

To study the interactions of mature BilRI with a human macrophage cell line, the *bilRI* gene was cloned into the pET-15b vector (Novagen), and the recombinant protein was produced as described previously [24]. Since we aimed to use the mature form of BilRI without any tags or lipid components, after binding to a 5-ml HisTrap HP (GE Healthcare, Uppsala, Sweden) column [36], recombinant BilRI containing an N-terminal 6-histidine tag was digested with 200 NIH units of thrombin (MP Biomedicals, Santa Ana, CA, USA) at room temperature overnight. The released BilRI was eluted with binding buffer (20 mM NaH_2_PO_4_/Na_2_HPO_4_, 800 mM NaCl, 20 mM imidazole, pH 7.5), and purification was continued with size exclusion chromatography as described for His-tagged BilRI [36].

The production and purification of ^15^N,^13^C-labeled BilRI for NMR studies has been described in Tossavainen et al. 2020 [37].

## NMR spectroscopy for the structural description of BilRI

The chemical shift assignment performed for BilRI has been described elsewhere [37]. Heteronuclear NOE, T_1_ and ^1^H, ^15^N NOESY-HSQC data were acquired at 25°C on a Bruker AVANCE III HD 800 MHz spectrometer equipped with a TCI ^1^H/^13^C/^15^N cryoprobe and a z-gradient coil. {^1^H}-^15^N heteronuclear NOE values were calculated as the intensity ratios of peaks from a pair of spectra measured with and without ^1^H presaturation during the recycle delay. The relaxation delay was set to 10 s. T_1_ relaxation delays of 20, 100, 200, 400, 600, 800, 1100, and 1400 ms were used. The recycle delay was set to 2.5 s. The mixing time in the NOESY-HSQC spectrum was set to 300 ms. NMR data were processed with TopSpin 3.5 (Bruker) and analyzed with CcpNmr Analysis v. 2.4.2 [38].

For BilRI-LPS interaction studies, LPS was extracted from the *A. actinomycetemcomitans* D7S Δ*flp1-flp2::spe* mutant [24]. The LPS concentration in the extract was approximately 10 mg/ml (1×10^6^ EU/ml; *Limulus* amoebocyte lysate (LAL) assay), and it contained impurities of 4.8 μg/ml protein and 145 μg/ml DNA. Since the purified LPS contained small amounts of DNA, we tested whether BilRI interacts with DNA by using an electrophoretic mobility shift assay [18].

## Bacterial strains and growth conditions

*A. actinomycetemcomitans* clinical strain D7S (serotype a) [39,40] was used as the wild-type variant in the experiments. A markerless mutant devoid of the *bilRI* gene was constructed from the D7S strain in our earlier study [30]. The strains were stored as stocks in either milk or culture medium supplemented with glycerol (20%) at −80°C and were revived by cultivation on tryptic soy agar (TSA) (37 g/L tryptic soy agar, 3 g/L agar) containing 5% defibrinated sheep blood.

## Human serum collection

Human venous blood was collected as described previously [36] from *A. actinomycetemcomitans*-positive adult periodontitis patients (21) and healthy controls (13) who provided written informed consent. Collection was conducted by a laboratory nurse at the Community Dental Health Care Center of Turku (Institute of Dentistry, University of Turku) or by a physician at the Unit for Specialized Oral Care in the Helsinki Metropolitan Area and Kirkkonummi (Helsinki, Finland).

## Screening of *A. actinomycetemcomitans* and BilRI-specific antibodies

Specific antibodies against *A. actinomycetemcomitans* and its OMP BilRI were screened using a microwell-based assay as described in detail for another OMP of *A. actinomycetemcomitans*, emHofQ [36]. Briefly, the wells were coated with an *A. actinomycetemcomitans* suspension containing equal amounts of strains D7S Δ*flp1-flp2::spe* (a), S23A (b), NCTC9710 (c), SA492 (d), 173 (e) and Tr.GU 17–4 (f) or with recombinant BilRI (500 ng) [30]. After blocking with BSA-containing blocking buffer, 1/100 (anti-*Aa)* or 1/20 (anti-BilRI) dilutions of patient/control sera were added, followed by incubation at room temperature overnight. The bound human IgG antibodies were detected with an anti-human IgG (Fc specific) peroxidase-coupled antibody (Sigma-Aldrich, A0170) using 2.2´-azino-bis(3-ethylbenzothiazoline-6-sulfonic acid) diammonium salt (Sigma-Aldrich, A9941) as the substrate. The wells containing *A. actinomycetemcomitans* cells were additionally blocked with BSA for 10 min before adding the secondary antibody. The results were obtained from 21 patient sera and 13 control sera. Nonspecific binding to the blocking agent BSA was subtracted from the results obtained for *A. actinomycetemcomitans* or BilRI binding, sometimes resulting in negative values.

## Measuring the induction of ROS production in isolated human leukocytes

ROS production by isolated human leukocytes was investigated using a chemiluminescence-based assay [41] after the leukocytes were challenged with either the wild-type D7S strain or its Δ*bilRI* mutant, as described previously in a study examining the induction of ROS production in Δ*hofQ* mutants of *A. actinomycetemcomitans* [36]. Briefly, bacteria were mixed with human serum in a buffer solution containing luminol to enhance the chemiluminescence reaction. Light production from the reaction was measured every 2 min immediately after adding the freshly isolated leukocytes. The leukocytes came from one healthy individual. The peak values from each reaction were recorded and compared.

## Macrophage stimulation

THP-1 human acute monocytic leukemia cells (ATCC® TIB-202™) [42] were cultured in RPMI-1640 medium (Gibco) supplemented with 10% FBS (Sigma) and 100 U/ml penicillin-streptomycin (#P4333, Sigma) at 37°C under 5% CO_2_. All incubations were performed under these conditions unless otherwise stated.

THP-1 monocytes were differentiated into macrophages by adding 150 µl of a cell suspension containing 10^6^ cells to the wells of 96-well plates, followed by incubation with 50 nM phorbol 12-myristate 13-acetate (PMA) for 24 h. After differentiation, the cells were washed with 150 µl PBS (#D8537, Sigma) and incubated in fresh medium for an additional 24 h. Stimulation agents (recombinant BilRI and LPS from *A. actinomycetemcomitans* [43]) were diluted in medium at a concentration of 0.1 ng/ml and incubated at RT for 1 h. The cells were washed with PBS, and 150 µl of stimulation agent-containing medium was added to the wells. After 20 h of incubation, the medium was collected and stored at −20°C. IL-6 concentrations were measured in the medium samples with a Single-Analyte ELISArray Kit (Qiagen).

Cell viability was measured immediately after medium collection using a neutral red uptake assay [44]. Briefly, the cells were washed with 150 µl PBS and incubated with 100 µl of 40 µg/ml neutral red dye diluted in RPMI medium for 90 min. The cells were washed as previously described and destained with 150 µl lysis solution (EtOH:MQ:acetic acid at 50:49:1 (v/v)) for 30 min at RT. The amount of released dye was detected by measuring the absorbance at 540 nm.

## Antimicrobial susceptibility testing

The antimicrobial susceptibility of the *A. actinomycetemcomitans* D7S wild-type and Δ*bilRI* mutant strains was tested using Etest® strips (BioMerieux) as described earlier [36]. The CLSI antimicrobial susceptibility testing breakpoint table (M100, 2017, table for *H. influenzae*) was used when interpreting MIC values. The mean MIC values from 5 to 9 independent experiments were calculated.

## Investigation of the natural transformation efficiency

The effect of the *bilRI* deletion mutation on the efficiency of DNA uptake from the surrounding environment by *A. actinomycetemcomitans* strain D7S (wild type), the markerless *bilRI* deletion mutant strain (Δ*bilRI*) [30], and a mutant strain in which the spectinomycin resistance cassette was substituted for *bilRI* (Δ*bilRI::spe*^r^ [30]) was examined by performing natural transformation using a linear DNA construct comprising an antibiotic resistance cassette flanked by DNA sequences complementary to the genomic DNA of *A. actinomycetemcomitans*. Blunt-end DNA sequences were used for natural transformation, which was performed by following the method described by [39] with some modifications, as described in detail in the Supplemental Materials and Methods.

The assay was repeated five times, and each experiment included two to three replicate samples from each strain. For each experiment, the average number of transformants in the Δ*bilRI*-strain and the Δ*bilRI::spe*^r^-strain was normalized against the average number of transformants in the wild-type strain. The statistical significance of the observed differences in the transformation efficiency between the different strains was analyzed using the Mann–Whitney U-test (IBM SPPS Statistics 26).

## Cloning of the *dsred* gene and expression of red fluorescence protein (RFP) in *E. coli*

The synthetic *dsred-M1* gene, flanked by 5´-terminal XhoI and 3ʹ-terminal EcoRI and SalI restriction sites (*dsred^E.c^*) with optimized codon usage for *E. coli* expression (Table 1), was ordered from Eurofins Genomics. The *dsred^E.c^* gene was digested from the cloning vector with XhoI and BamHI (at sites originating from the vector) and ligated into the pET-15b vector (Novagen, Darmstadt, Germany) using T4 DNA ligase (Thermo Fisher Scientific), and the inserted region was sequenced (Eurofins Genomics, Germany) before being used for *E. coli* expression. For the expression of RFP, the pET-15b_dsred^E.c^ plasmid was transformed into the *E. coli* BL21 CodonPlus (DE3)-RIL (Stratagene, San Diego, CA, USA) protein expression strain.

**Table 1.** Codon-optimized genes used for protein expression. Restriction enzyme sites are underlined

Gene name	Codon-optimized sequence
*dsred^E.c^* *(the last 24 nucleotides originated from the cloning vector)*	CTC GAG ATG GAC AAT ACC GAG GAC GTG ATT AAG GAG TTC ATG CAG TTT AAA GTG CGC ATG GAA GGG TCT GTC AAT GGG CAC TAT TTC GAG ATT GAA GGC GAA GGT GAA GGC AAA CCG TAT GAA GGC ACA CAG ACC GCC AAA CTG CAA GTG ACA AAA GGT GGT CCT TTA CCG TTT GCG TGG GAT ATC CTT TCA CCC CAG TTT CAG TAT GGA TCG AAA GCG TAT GTC AAA CAT CCC GCA GAT ATT CCG GAC TAC ATG AAA CTC TCG TTT CCG GAA GGC TTT ACT TGG GAA CGC TCC ATG AAC TTT GAG GAT GGA GGT GTA GTG GAA GTA CAG CAA GAC AGC TCT CTG CAA GAT GGC ACG TTT ATC TAC AAA GTC AAA TTC AAA GGT GTA AAC TTC CCA GCT GAT GGT CCG GTT ATG CAG AAG AAA ACT GCA GGC TGG GAA CCT AGC ACC GAG AAA CTG TAT CCG CAA GAT GGC GTT CTG AAA GGC GAA ATT AGT CAT GCG TTG AAA CTG AAA GAT GGC GGT CAT TAC ACG TGC GAT TTC AAA ACC GTC TAC AAA GCC AAG AAG CCG GTT CAA TTA CCA GGG AAT CAC TAT GTG GAT AGT AAG CTG GAC ATC ACG AAC CAC AAC GAG GAC TAT ACC GTT GTG GAA CAG TAC GAA CAT GCC GAA GCT CGT CAT AGC GGT TCC CAG TAA GAA TTC GTC GAC *ACC TGC TTT TGC TCG CTT GGA TCC*
*dsred^A.a^*	GGT ACC ACA ATT GTT AAT TTA GGA GTA ACG ATG GAC AAT ACG GAG GAT GTC ATC AAG GAG TTC ATG CAG TTC AAA GTA CGC ATG GAA GGC TCC GTC AAT GGC CAC TAC TTC GAA ATT GAG GGT GAG GGT GAA GGC AAA CCG TAT GAA GGA ACT CAG ACA GCG AAA CTT CAG GTG ACC AAA GGT GGT CCA CTC CCG TTT GCT TGG GAC ATT TTG TCT CCG CAA TTT CAG TAT GGC TCT AAA GCC TAC GTG AAA CAT CCT GCG GAC ATT CCG GAC TAC ATG AAA CTG TCG TTT CCC GAA GGG TTT ACG TGG GAA CGC TCC ATG AAC TTT GAG GAT GGT GGC GTT GTA GAA GTC CAA CAA GAC AGT AGC TTA CAG GAT GGC ACC TTC ATC TAC AAG GTG AAA TTC AAA GGT GTC AAC TTT CCC GCA GAT GGT CCA GTA ATG CAG AAG AAA ACG GCT GGA TGG GAA CCG AGC ACT GAG AAG CTG TAT CCG CAA GAT GGC GTT CTG AAA GGC GAA ATT TCG CAT GCC CTG AAA CTG AAA GAT GGT GGG CAC TAT ACA TGC GAC TTT AAA ACC GTG TAC AAA GCC AAG AAA CCG GTT CAG TTA CCT GGG AAC CAC TAT GTG GAT AGC AAA CTG GAT ATC ACC AAT CAT AAC GAA GAT TAT ACC GTT GTG GAA CAG TAT GAA CAT GCA GAA GCG CGT CAT AGT GGC TCA CAA TAA GTC GAC

For the expression and purification of mature RFP from the BL21_dsred^E.c^ strain, the cells were grown in 2 x TY-medium (16 g/L tryptone, 10 g/L yeast extract, 5 g/L NaCl) supplemented with 100 μg/mL ampicillin at 30°C to an OD_600nm_ of ~1, after which protein expression was induced by 1 mM IPTG. Growth was continued at 30°C overnight, and the cells were then collected by centrifugation at 6000 × g for 10 min at 4°C. The cells were lysed by sonication (10–12 μm amplitude, 4 × 30 s on ice) in buffer A (20 mM Na_2_HPO_4_, 300 mM NaCl, 20 mM imidazole, pH 7.5) with an additional 1 μg/mL DNaseI (Thermo Fisher Scientific), 10 mM MgCl_2_ and 0.2 mM phenylmethylsylfonyl fluoride (PMSF, Sigma) as protease inhibitors. The lysate was centrifuged at 48 000 × g for 30 min at 4°C, and the supernatant was then filtered through a 0.45 μm filter membrane (Sarstedt). The lysate was then loaded into a PD-10 gravity flow column filled with ~3 mL TALON Super Flow cobalt matrix (GE Healthcare). Prior to loading the lysate, the matrix was rinsed with 10 mL MQ to remove the preservative ethanol and balanced with 20 mL buffer A. After loading the lysate, the matrix was rinsed with 15 mL buffer A. The pET-15b-expression vector inserts a 6-histidine-long tag and a thrombin cleavage site into the N-terminal end of RFP. To release the RFP that was bound to the matrix, the filtrate was digested with 200 units of thrombin (MP Biomedicals, Santa Ana, CA, USA) dissolved in buffer A in a volume sufficient to cover the matrix. The column was then shielded from light with aluminum foil, and the cap was installed to prevent dehydration. Thrombin digestion was performed at ambient temperature overnight. The volume in the column (~3 mL) was then collected, and the presence of digested RFP could be visually verified from the strong red color of the eluate, which faded as PBS was added to elute the remaining contents in the column. The concentration of RFP in the red eluate was determined via the Lowry method [45], and the sample was stored at −20°C.

**Cloning of the *dsred* gene and expression of RFP under the genomic *bilRI* promoter in *A. actinomycetemcomitans***

To study the activity of the promoter region of the *bilRI* gene, the gene encoding *bilRI* (NC_017846.1 AaD7S_02241) was replaced with the *dsred-M1* gene, preserving the promoter region of *bilRI*. The *dsred-M1* gene was introduced into *A. actinomycetemcomitans* via the natural transformation of a linear DNA construct that contained the sequence for the *dsred-M1* gene followed by the sequence for the spectinomycin resistance cassette. At both ends of the construct, sequences flanking the *bilRI* gene were inserted in both upstream and downstream directions. Synthetic DNA for *dsred-M1*, flanked by 5´-terminal KpnI and MfeI, and 3´-terminal SalI restriction sites (*dsred^A.a^*) with optimized codon usage for *E. coli* expression (Table 1), was ordered from Eurofins Genomics. The construction of genetically modified *A. actinomycetemcomitans* is described in more detail in the Supplemental Materials and Methods.

## Screening of RFP expression in *A. actinomycetemcomitans* under various growth conditions

The expression of RFP in the *ΔbilRI::dsred* mutant strain of *A. actinomycetemcomitans* was initially screened by measuring the fluorescence signal intensity in the cells growing as biofilms in 96-well plates (data not shown). For this purpose, suspensions of plate-grown (TSA, 3–4 days) cells were prepared in TSB (supplemented with glucose), and 2–3 × 10^7^ colony-forming units (CFUs) per well were plated on 96-well plates. The plates were incubated at 37°C in candle jars for 24 h for bacterial growth, after which the growth conditions in some of the wells were modified, and the plates were grown for an additional 24 h or longer. Fluorescence intensity measurements were performed on a Hidex Sense microplate reader (Hidex Oy, Turku, Finland) using 544 nm excitation and 595 nm emission filters. The growth medium was removed from the biofilms, and the wells were rinsed with PBS prior to fluorescence measurements.

For the growth conditions that appeared promising in the 96-well plate format, larger- cale studies were performed by growing 1.2 × 10^8^ CFU/mL of the *ΔbilRI::dsred* mutant strain in 5 mL of TSB (supplemented with glucose) in cell culture bottles (Cellstar® Cell culture flasks, 50 ml, Greiner bio-one, Germany) or 1.44 × 10^9^ CFUs of the strain on TSB-agar plates (supplemented with glucose) at 37°C in candle jars. After 24 h of growth, new growth conditions were implemented. The cells were grown for an additional 72 h and were finally scraped out the culture bottles and centrifuged at 4000 x g for 5 min at 4°C. The cell pellet was then rinsed with MQ and centrifuged at 16,000 x g for 4 min at 4°C. The cells were then lysed in MQ by sonication (10 μm amplitude, 3 × 10 s on ice) and centrifuged at 16,000xg for 2 min at 4°C. When high protein concentrations were required, the MQ-based supernatants were concentrated with an Eppendorf Concentrator Plus (Eppendorf AG, Hamburg, Germany). The protein concentration in the lysed biofilm samples was determined using the Lowry method [45]. The protein samples were then run on a polyacrylamide gel (10% Criterion TGX stain free protein gel, Bio-Rad) under native conditions using Tris-glycine as the running buffer. DsRed was detected via in-gel fluorescence detection with the Chemidoc MP Imaging System (Bio-Rad). DsRed signal intensities under the tested growth conditions were normalized against the appropriate control conditions, and statistical significance was analyzed using the Kruskal–Wallis test and the post hoc Mann–Whitney U-test with Bonferroni correction.

## Results

### Highly conserved BilRI shows sequence homology to LEA proteins

The *bilRI* gene could be found in every sequenced *A. actinomycetemcomitans* strain deposited in GenBank. Its sequences were highly conserved at the amino acid level, showing over 95% sequence similarity in most cases (Fig. S1). The protein variants of all strains were roughly divided into five clusters based on amino acid similarity (Fig. S2). However, the changes did not alter the properties of the side chain in the majority of the cases, meaning that the hydrophobicity/hydrophilicity of the side chain remained unaltered. The grand average of hydropathicity index (GRAVY) [46] was −0.707 for the mature BilRI protein of *A. actinomycetemcomitans* D7S, indicating that it is highly hydrophilic.

**Figure 1.**
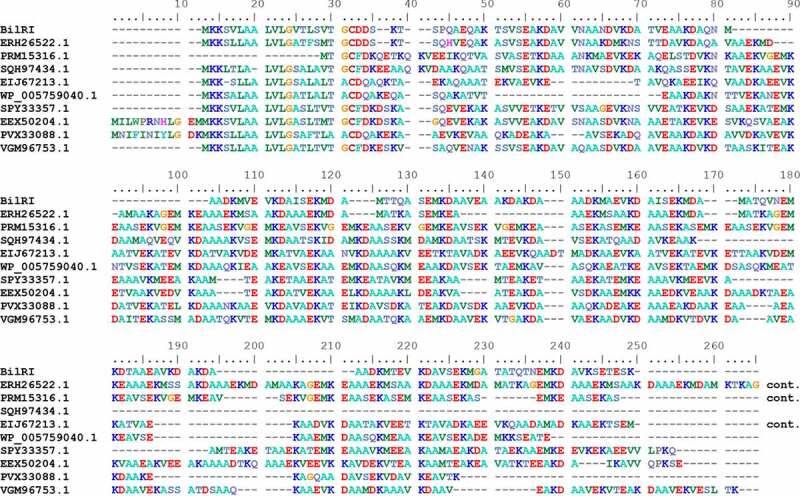
*A. actinomycetemcomitans* D7S BilRI shares high amino acid sequence similarity with various LEA proteins from gram-negative species belonging to *Pasteurellaceae.*

BLAST searches revealed sequence homology between *A. actinomycetemcomitans* D7S BilRI and various other LEA proteins from bacteria (Figure 1) and plants (Fig. S3). BilRI shared greater sequence homology with other bacterial LEA proteins than with plant LEA proteins. The bacterial LEA proteins all came from gram-negative species belonging to the family *Pasteurellaceae* except for one gram-positive species, *Gemella haemolysans*, whose sequence (EER68557.1) was ultimately excluded from the alignment because it was significantly longer than the rest of the sequences. All bacterial LEA proteins belonged to the LEA_4 protein family [47] according to Pfam classification based on the sequence motifs present in the proteins (LEA_4: PF02987). This group of proteins is also referred to as LEA group 3 or D-7/D-29 [48,49], but in this paper, we will follow the Pfam nomenclature. Most of the plant LEA proteins consisted of either embryogenic cell protein 63 (ECP63) or At3g53040 sequences from different species (Fig. S3). Both ECP63 and At3g53040 belong to the LEA_4 protein family. LEA_4 proteins share a characteristic repeating 11-mer motif (reviewed in [1,48,49], and the protein sequence is reported to exhibit regularly repeating lysine (K), aspartic/glutamic acid (D/E) and alanine (A) residues [48], which can also be found in the sequence of *A. actinomycetemcomitans* BilRI (Fig. S1). BilRI contains eleven 11-mer repeats, ten of which end in KD[A/T].

## Structural description of BilRI

The low complexity of the amino acid sequence of BilRI, which included no aromatic residues but contained a pronounced number of charged and polar residues, strongly suggested that BilRI is intrinsically disordered; indeed, the ^1^H and ^1^H, ^15^N HSQC spectra clearly demonstrated features typical of an IDP [30]. We further characterized its structural features by analyzing the recently assigned chemical shifts of BilRI [37] and its {^1^H}-^15^N heteronuclear nuclear Overhauser effect (NOE), T_1_ and ^1^H, ^15^N nuclear Overhauser effect spectroscopy-heteronuclear single quantum coherence spectroscopy (NOESY-HSQC) spectra.

**Figure 2.**
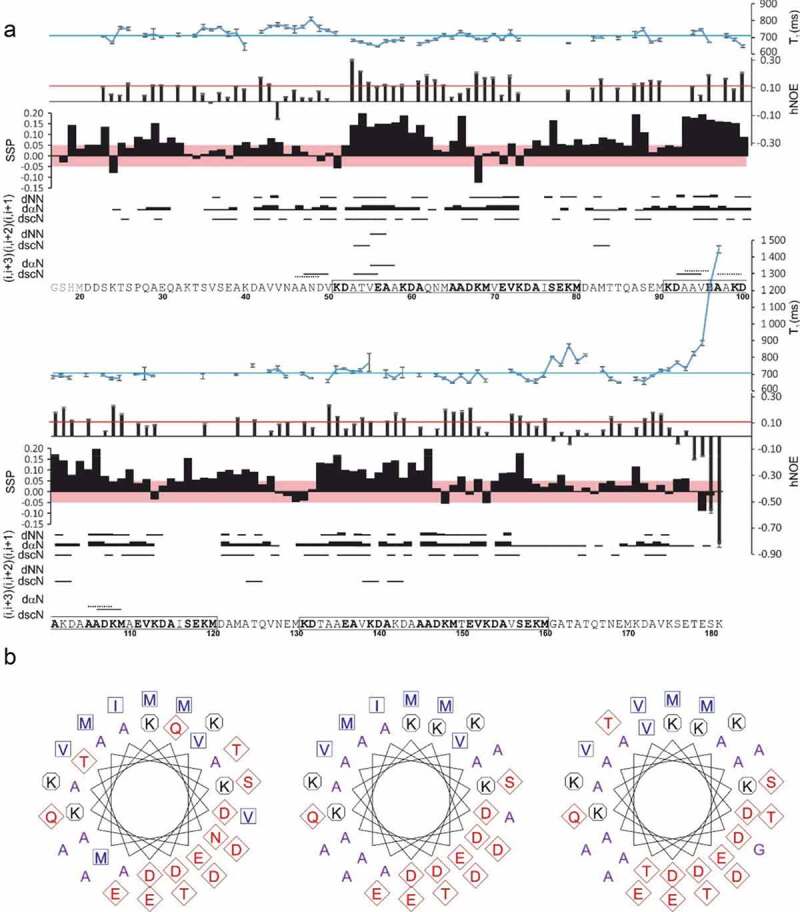
(a) Structural characterization of BilRI according to T_1_ and heteronuclear NOE (hNOE) results, secondary structure propensity derived from the assigned chemical shifts and short-range NOE correlations. T_1_ is shown at the top, in which the average value is indicated with a blue line (708 ms), whereas the red line indicates the average hNOE value (0.10). The secondary structure propensity (SSP) score ranges from +1 to −1 for a fully formed helix and strand, respectively. For short-range NOEs, dNN, dαN and dscN refer to the observed H^N^-H^N^, H^α^-H^N^ and side chain-H^N^ NOE correlations, respectively, and the width of the bar represents the relative intensity of the peak. The three repeats in the amino acid sequence are boxed, and identical residues are highlighted in bold. The first four residues of the studied protein are a cloning artifact. (b) Helical wheel representations of the repeating segments (51–80, 91–120, 131–160) in the BilRI amino acid sequence. The Figure was generated on the EMBOSS pepwheel web server (http://www.bioinformatics.nl/cgi-bin/emboss/pepwheel)

Chemical shift assignment for BilRI was extremely complicated due to the presence of repeating segments in the amino acid sequence leading to considerable overlap of NMR peaks [37]. This overlap also hampered the analyses of ^15^N relaxation and NOESY spectra because of the crowded ^1^H, ^15^N HSQC spectrum. Nevertheless, a fair amount of data was retrieved (Figure 2(a)).

The chemical shifts of H^α^, C^α^, and C’ are particularly sensitive to the ϕ/ψ angles of the backbone, making them good probes for any residual secondary structure in an IDP. Secondary H^α^, C^α^, and C’ chemical shifts were tabulated by comparison with temperature-, pH- and neighbor-corrected random coil chemical shifts generated using POTENCI [50] and were combined with a single SSP score similar to that described by Marsh and coworkers [51]. The deviations of the experimental BilRI chemical shifts from their random coil values were small (−0.13< SSP <0.23) throughout the sequence and were biased toward helical propensity (positive SSP scores). The score pattern was similar within the three repeating segments of the sequence (51–80, 91–120, and 131–160), suggesting that the minute residual structural propensity is not random: the N-terminal residues of these repeats show a helical tendency.

It is rather surprising that BilRI exhibits such low experimental helicity, which is in clear conflict with the secondary structure predictions based on its amino acid sequence. The PSIPRED and JPred4 servers [52,53] predicted helical secondary structure with high confidence for 97 and 92% of the BilRI residues, respectively. s2D software, which simultaneously predicts intrinsic disorder and secondary structure [54], predicted a > 50% helical secondary structure population among 81% of BilRI residues. The three repeats present in the BilRI sequence exhibit regularly distributed hydrophobic residues and could thus theoretically form amphipathic helices (Figure 2(b)). We speculated that helicity in BilRI might build-up in an environment more closely resembling its cellular milieu, possibly due to folding induced by binding to a natural partner. We tested this hypothesis in an NMR titration assay in which we monitored changes in the ^1^H,^15^N HSQC spectrum of BilRI following the addition of increasing concentrations of LPS (Fig. S4). We found that while LPS (approximately 2 mg/ml) clearly interacted with BilRI, it did so via residues located outside of the BilRI’s helical repeats. As the peaks arising from residues in the repeats remained unchanged in the titration spectra, we deduced that LPS has no effect on the helicity of the repeats. Since purified LPS contained small amounts of DNA, we ensured that BilRI did not interact with DNA (data not shown).

While the chemical shift data suggested a low propensity for secondary structure formation, they did not rule out the possibility of tertiary contacts within the BilRI molecule. To examine this possibility, we acquired a 3D NOESY-^1^H, ^15^N HSQC spectrum, which reveals the presence of local structures as NOE peaks between distant residues within the amino acid sequence. Sequential H^α^(i)-H^N^(i + 1) NOE peaks were found for most residues (Figure 2(a)). Abundant H^N^(i)-H^N^ (i + 1) and side chain(i)-H^N^(i + 1) correlations were also observed, although with lower intensities. The simultaneous presence of sequential αN and NN NOEs is explained by the presence of both α and β conformers in the ensemble [55]. In addition, a few i, i + 2/i + 3 NOEs were present. These are typically observed in helical structures. In BilRI, these NOEs were predominantly observed in regions with significantly positive SSP scores, i.e., helices. However, the identification and assignment of these peaks was notably difficult because of the overlap, and the total number of such peaks remains uncertain. No long-range NOEs were observed. Transient long-range structures could also be observed in the backbone dynamics of the protein. We therefore acquired ^15^N T_1_ and {^1^H}-^15^N heteronuclear NOE (hNOE) spectra. These spectra provide a measure of backbone flexibility on a pico-nanosecond timescale. The average T_1_ and hNOE values were 708 ± 40 ms and 0.10 ± 0.07, respectively. For comparison, a well-structured, globular protein of similar size shows T_1_s of ~1100 ms and hNOEs >0.8 at an 800 MHz ^1^H spectrometer frequency at 25°C. The T_1_ and hNOE values were relatively uniform within the repeats, with slightly lower average T_1_s and higher hNOEs than were found in the rest of the molecule. Gly161 likely contributes to the faster internal motion of residues 160–167 [56] and, through slower cross-relaxation rates, to the sparseness of NOE peaks for residues 158–171. The C-terminal “end effect” is commonly observed at protein termini and arises from a high degree of flexibility of terminal residues. Overall, the BilRI backbone appears to be very flexible, as expected, and there is no evidence of specific long-range contacts in BilRI. The repeat regions, especially their N-terminal 2/3, appear to be slightly more rigid than the rest of the protein.

## BilRI does not elicit antibody production in periodontitis patients

**Figure 3.**
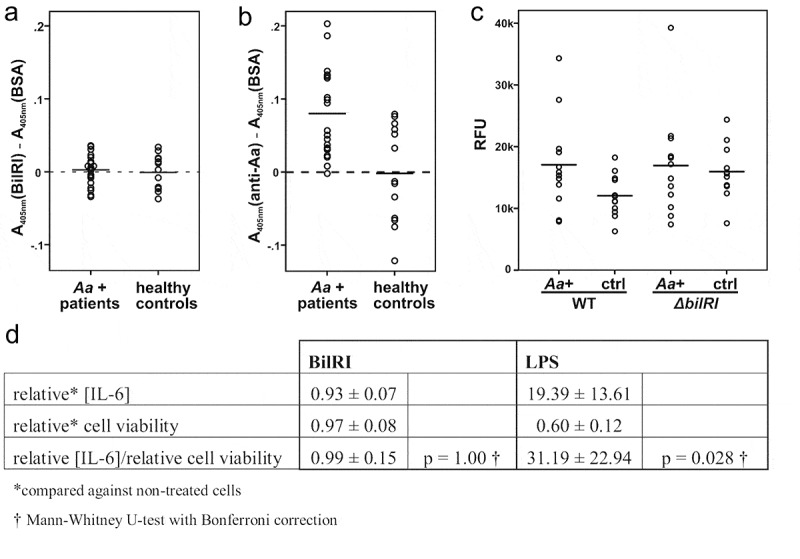
BilRI is not involved in interactions between *A. actinomycetemcomitans* and human immune cells. (a) BilRI did not stimulate antibody production in *A. actinomycetemcomitans* culture-positive periodontitis patients, although (b) *A. actinomycetemcomitans-*specific antibodies could be found in these patients. (c) A deletion mutant of *A. actinomycetemcomitans* devoid of the *bilRI* gene showed stimulation of neutrophil ROS production as efficiently as the wild-type strain when opsonized with either *A. actinomycetemcomitans* culture-positive patient sera (*Aa*+) or healthy control sera (ctrl). (d) Recombinant BilRI did not stimulate IL-6 production from human THP-1 macrophages and was nontoxic to the cells, whereas *A. actinomycetemcomitans* LPS induced high IL-6 production and decreased cell viability

An earlier study involving *A. actinomycetemcomitans* OM lipoprotein PAL (AaPAL) indicated that AaPAL is immunoreactive, especially among periodontitis patients [14]. Therefore, we wanted to examine whether BilRI, which is a lipoprotein located in the OM, similar to AaPAL [29], could also elicit strong antibody production in periodontitis patients who harbor *A. actinomycetemcomitans*. The levels of BilRI-specific antibodies did not differ significantly between *A. actinomycetemcomitans*-positive periodontitis patients and healthy controls (p = 0.710; Mann–Whitney U-test) (Figure 3(a)). Moreover, the mean absorbance value in the BilRI-specific antibody measurements was close to zero (Figure 3(a)), whereas significant levels of *A. actinomycetemcomitans-*specific antibodies could be measured in the sera of *A. actinomycetemcomitans*-positive periodontitis patients (p = 0.007; Mann–Whitney U-test) (Figure 3(b)).

## BilRI is not involved in the induction of ROS production by leukocytes

Since BilRI faces the extracellular space [29], it might be involved in interactions with host cells. To elucidate these potential interactions, we examined whether there was a difference between the wild-type cells and the Δ*bilRI* mutants regarding the potential for the induction of reactive oxygen species (ROS) production by leukocytes. When opsonized with sera from *A. actinomycetemcomitans*-positive periodontitis patients, the *A. actinomycetemcomitans* wild-type cells showed the induction of ROS production to a slightly higher level that that observed following opsonization with sera from healthy controls (Figure 3(c)); however, the difference was not statistically significant (p = 0.068; Mann–Whitney U-test). Moreover, similar results were obtained with the Δ*bilRI* mutant, suggesting that BilRI does not play a role in bacteria-leukocyte interactions (Figure 3(c)).

## The recombinant BilRI backbone does not induce interleukin-6 production by human macrophages

Interleukin (IL)-6 was selected as an indicator cytokine for BilRI stimulation studies in human THP-1 macrophages since *A. actinomycetemcomitans* cells and eDNA stimulate the production of IL-6 in mouse macrophages in a Toll-like receptor (TLR) 2- and TLR4-dependent manner [57]. Moreover, a recent systematic review indicated that IL-6 might be used as a diagnostic salivary biomarker of periodontitis [58]. Although *A. actinomycetemcomitans* LPS, which was used as a positive control, caused significant IL-6 production in THP-1 macrophages after 20 h of stimulation (p = 0.028, Mann–Whitney U-test with Bonferroni correction), the results obtained with the recombinant 165 amino acid-long BilRI polypeptide did not differ from those in the control experiment without any stimulation (p = 1.00 Mann–Whitney U-test with Bonferroni correction) (Figure 3(d)).

## Δ*bilRI* mutants show similar antibiotic susceptibility to the parental wild-type *A. actinomycetemcomitans* strain

**Figure 4.**
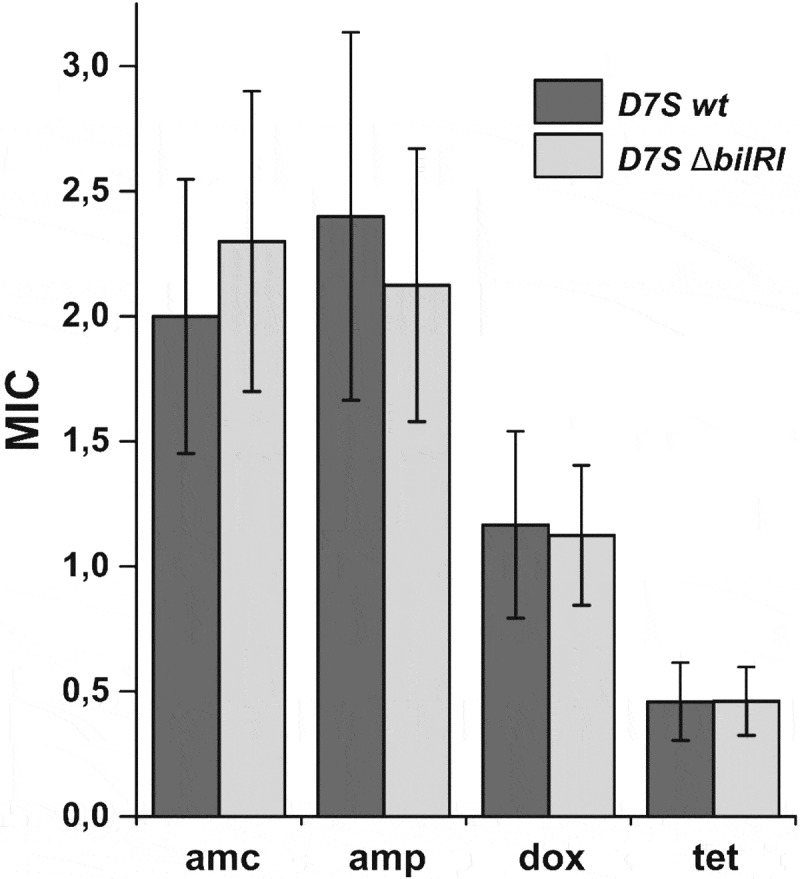
The Δ*bilRI* deletion mutant shows similar susceptibility to various antibiotics to that of the wild-type D7S strain. MIC: minimal inhibitory concentration; amc: amoxicillin-clavulanic acid; amp: ampicillin; dox: doxycycline; tet: tetracycline

The components of the OMs of gram-negative species may protect the bacteria from various antibiotics. Therefore, we investigated whether the deletion of the *bilRI* gene changes the profile of *A. actinomycetemcomitans* susceptibility to clinically relevant antibiotics. The wild-type strain and the Δ*bilRI* mutant strain were both susceptible to the tested antimicrobials, and *bilRI* deletion did not cause any changes in susceptibility to the beta-lactams penicillin and amoxicillin/clavulanic acid or the tetracycline-group antibiotics tetracycline and doxycycline (Figure 4).

## The stand-alone *bilRI* gene is located at the other end of the competence locus

**Figure 5.**
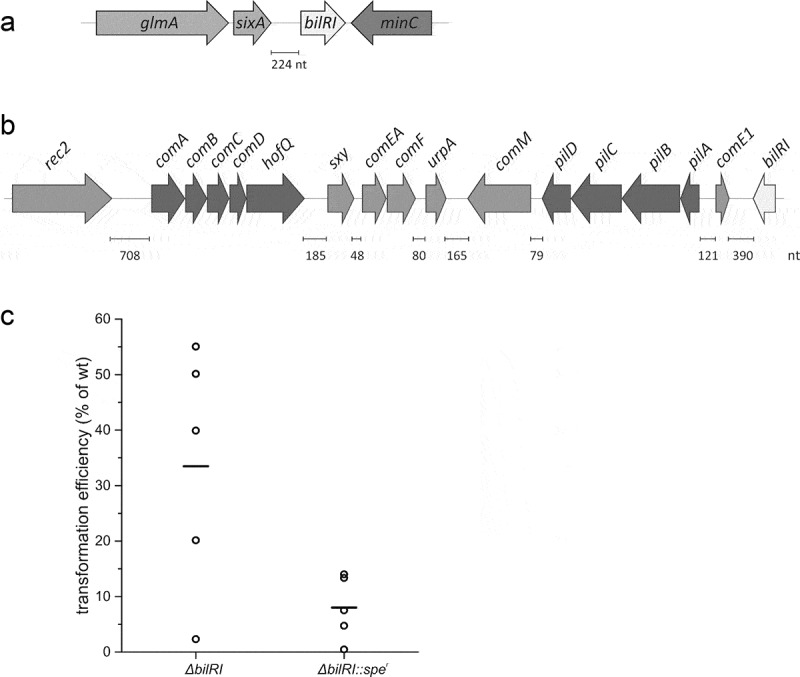
(a) The stand-alone gene *bilRI* (b) located near genes involved in natural transformation. (c) Δ*bilRI* mutants showed a lower transformation efficiency than the parental wild-type D7S strain

Since the location of the stand-alone *bilRI* gene (Figure 5(a)) [30] could predict its function, we further investigated its location in the *A. actinomycetemcomitans* D7S genome. The distance from *bilRI* to the nearest competence gene, *comE1*, was approximately 390 bp; however, these genes were located in opposite directions (Figure 5(b)). The closest competence genes in the same orientation were *pilA-D*, but these genes, like *bilRI*, most likely have their own promoters [19].

## Δ*bilri* mutants exhibit a lower transformation efficiency than the parental wild-type strain

Since *A. actinomycetemcomitans* clinical isolate D7S is naturally competent (*i.e*., it has the ability to take up eDNA and incorporate it into its genome) [40] and the *bilRI* gene is located in close proximity to the genes related to natural transformation, we wanted to investigate whether the OMP BilRI plays a role in this process. The deletion of *bilRI* dramatically decreased the transformation efficiency compared to that of the wild-type strain (p = 0.011) (Figure 5(c)). Since the added linear DNA was targeted to the site in the genome where the *bilRI* gene was deleted, we used two different Δ*bilRI* mutants in these experiments: one was markerless, and the other contained an *spe* gene, which replaced the *bilRI* gene [30]. The second mutant better mimicked the size of the wild-type locus, where homologous recombination should take place. Similar decreases in the transformation efficiency were seen in the two Δ*bilRI*-mutants (Figure 5(c)).

## BilRI expression increases at low temperatures

**Figure 6.**
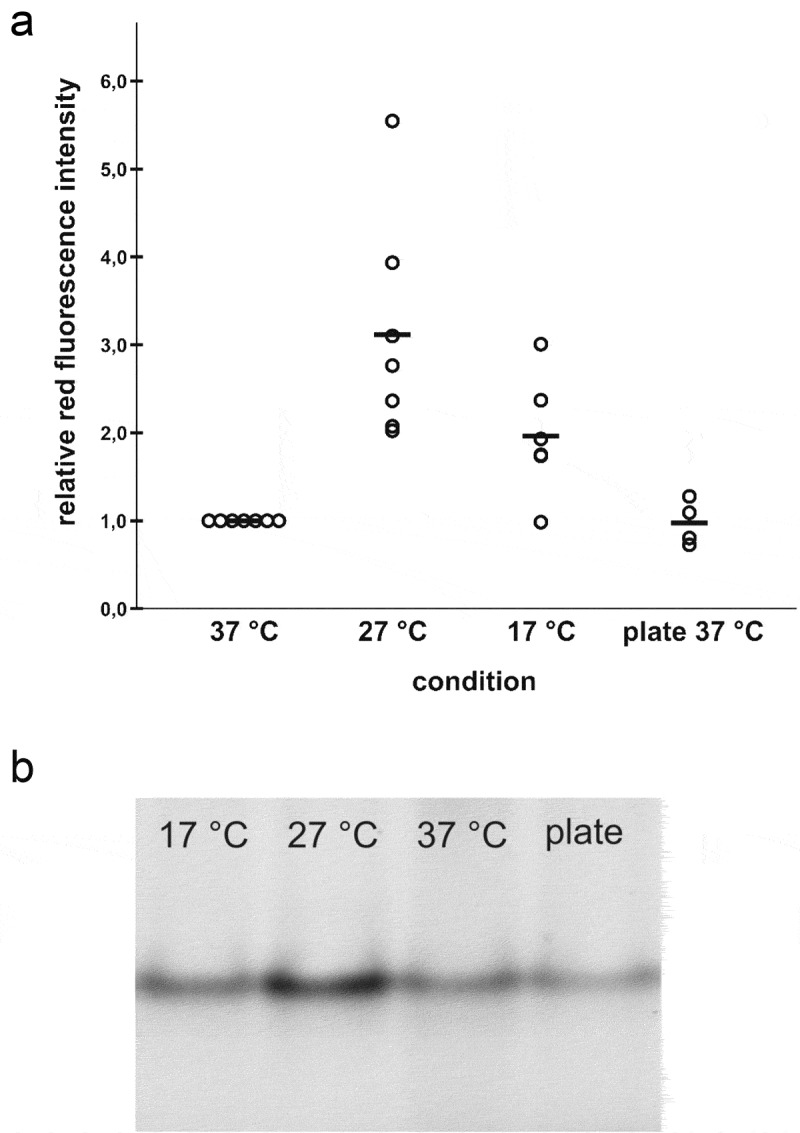
**The *bilRI* promoter is activated at reduced temperatures**. (a) The production of the red fluorescence protein DsRed-M1 was enhanced at reduced temperatures when its gene was cloned under the control of the genomic *bilRI* promoter in the *A. actinomycetemcomitans* D7S Δ*bilRI* mutant. Dry conditions, which were mimicked in plate culture, did not enhance production compared to that observed in biofilm culture in rich culture medium at the same temperature. (b) Relatively low levels of DsRed-M1 were expressed under all conditions. Approximately 60 ng of the total amount of protein loaded in each well (50 μg) consisted of DsRed-M1 at 27°C

Experiments conducted with DsRed as a reporter protein showed that the *bilRI* promoter was activated when the temperature was lowered from 37°C to 27°C (p = 0.002); however, a further decrease in temperature to 17°C did not increase the amount of DsRed protein to as great an extent (p = 0.089) (Figure 6). Approximately 2–3 times higher fluorescence of the DsRed reporter protein was observed after incubation at 27°C versus incubation at 37°C for three days. The amount of DsRed accounted for approximately 0.1% of the total protein at 27°C. Various different conditions, such as 24 h of exposure to 0.5 M ethanol, 2% NaCl, 0.5% glycerol, and 0.5 M sucrose, were tested, but changes in expression levels were detected only at lower temperatures (data not shown). Since BilRI shared sequence homology with LEA proteins, which are known to protect plant cells in dry conditions, we further investigated expression levels in plate-grown bacteria, mimicking dry conditions compared to the ones in biofilms surrounded by liquid medium in cell culture bottles. The plate-grown *A. actinomycetemcomitans* cells showed similar expression levels of DsRed to the cell culture bottle-grown biofilms (p = 1.00) (Figure 6).

## Discussion

Various lines of evidence suggested that BilRI belongs to the LEA protein family. Mature BilRI exhibited a GRAVY index of −0.707, indicating that the protein is highly hydrophilic, a common characteristic of LEA proteins [2]. We previously found that BilRI is intrinsically disordered [30,37], and the more comprehensive NMR studies described in this paper reinforced these findings. In addition to the very small dispersion of peaks observed in HSQC spectra, BilRI exhibited the motional properties of an IDP; namely, it presented significantly lower T_1_s and hNOEs compared to a folded protein. The analysis of chemical shifts indicated a maximum 19–21% helix population within the three repeats. This is significantly lower occurrence than that found for IDPs with high helical propensity [59,60], but quite typical as compared to several other IDPs, e.g. Cancer testis antigen 16, cytoplasmic tail of adenosine receptor A2A, and α-synuclein [61–63]. BLAST searches revealed sequence homology between *A. actinomycetemcomitans* D7S BilRI and various LEA proteins, such as EPC63 and At3g53040, from plants. These proteins have been studied mainly in *Arabidopsis thaliana* (mouse-ear cress), in which ECP63 plays a role in seed maturation and desiccation tolerance [64] and the *at3g53040* gene has been reported to be more highly expressed in seeds that are in a dormant stage than in the after-ripening stage [65]. Expression also responds to abscisic acid (ABA); thus, At3g53040 might protect seeds against abiotic stress [66]. Bacterial LEA protein functions have not been extensively studied; however, some LEA_4 proteins play a role in resistance to abiotic stresses. DrLEA3 of *Deinococcus radiodurans* is involved in desiccation and oxidation tolerance [4,8], and Zmo0994 of *Zymomonas mobilis* increases tolerance to ethanol [9]. Some LEA proteins may adopt α-helical structures when water levels decrease [67], and the α-helical structure of dehydrins, which form a separate dehydrin class in the Pfam LEA classification, interacts electrostatically with lipid membranes [68]. The experimental NMR data indicated that BilRI exhibits low helicity, although the secondary structure predictions showed helical structures with high confidence. It is possible that in its natural location, bound to the outer membrane and surrounded by the extracellular matrix, BilRI adopts a different distribution of secondary structure populations. In principle, the three repeats in the BilRI sequence have the potential to form amphipathic helices that could interact with ligands or the matrix in a polarized manner. Alternatively, the three transiently populated helices of BilRI could form a helical bundle with a hydrophobic core with numerous apolar contacts established by the abundant AMV residues. Although the addition of LPS (approximately 2 mg/ml) to BilRI induced perturbations in several cross peaks, they were located outside of the helical region; hence, no indication of LPS-induced conformational changes or folding localized in the helical region was observed. Various LEA proteins, especially dehydrins, sequester water and protect cells from drying during anhydrobiosis [69]. In addition to desiccation, LEA proteins defend plant cells against harmful effects caused by cold [70]. Investigations of *bilRI* promoter activity under different conditions revealed that it was more active at 27°C than at 37°C.

The most pronounced effect of BilRI on the physiology of *A. actinomycetemcomitans* concerned the ability to undergo natural transformation (*i.e*., to take up linear eDNA and incorporation of it into the homologous site of the genome). Only competent strains can realize all stages of this process, starting from efficient internalization of the uptake signal sequence containing linear DNA and ending with homologous recombination. Competent strains exhibit functional Com (A, B, C, D, E, EA, F, E1), Pil (A, B, C, D) and Rec2 proteins that take part in DNA uptake, in addition to the ComM and UrpA proteins, which are also needed for transformation [15,17,19]. Moreover, the *pga* gene cluster has been shown to be involved in the development of competence in *A. actinomycetemcomitans* biofilm-forming cells [71]. The addition of stop codons in the middle of competent genes or other types of insertions can lead to a loss of competence [15]. The *bilRI* gene has been predicted to be a stand-alone gene with its own promoter region [30]. It is flanked by *sixA*, encoding phosphohistidine phosphatase, and *minC*, whose product is involved in determining the septum site. Despite its stand-alone nature, the close proximity to the *comE1* and *pilA-D* genes, which function in natural transformation [19], indicates a role of BilRI in the uptake of eDNA. However, it is also possible that the deletion of *bilRI* affects either the composition of OMPs or the surface properties of the *A. actinomycetemcomitans* OM, which in turn weakens the ability to transfer eDNA inside the bacterial cell.

Some *A. actinomycetemcomitans* OM lipoproteins, such as cytolethal distending toxin subunit A, do not elicit significant antibody production in periodontitis patients [72], whereas others, such as conserved AaPAL, are immunogenic in periodontitis patients [14] and most likely play an important role in the stimulation of host immune cells [73,74]. Our study suggests that the OM lipoprotein BilRI might not exhibit such functions in *A. actinomycetemcomitans* infection. The sera of *A. actinomycetemcomitans*-positive periodontitis patients did not contain specific antibodies against BilRI, although more *A. actinomycetemcomitans*-specific antibodies were detected in their sera than in those of the healthy controls, suggesting that BilRI is a nonimmunogenic OMP. We used recombinant BilRI, which did not possess the lipid component attached to the C-terminal cysteine in our studies, so that we could screen only antibodies targeting the polypeptide chain of BilRI. Although some studies claim that intrinsically disordered proteins may be poor antigens with a low affinity for binding partner molecules [75], a recent extensive study demonstrated their ability to act as efficient antigens showing high-affinity antibody–antigen interactions [76]. Moreover, the disordered epitopes consist of short linear sequences of approximately 10 amino acids in length, which are buried in binding paratopes in antibodies exhibiting intimate contact [76]. Disordered epitopes present a high frequency of hot-spot residues in which polymorphisms may lead to significant effects on binding affinity [76]. *A. actinomycetemcomitans* BilRI contains four individual 10 amino acid-long sequences that are repeated three times [29]. These repeats are not 100% identical and differ in one to four amino acids [29]. Whether this sequence variation within the repeated sequences affects the immunogenic potential of mature BilRI is currently not known but could be interesting to study.

Consistent with the nonimmunogenic properties of BilRI described above, BilRI was not observed to play a role in the *A. actinomycetemcomitans*-leukocyte/macrophage interaction when ROS and IL-6 production by leukocytes/macrophages was used as an indication of effective contact. However, opsonization with *A. actinomycetemcomitans-*positive periodontitis patient sera (which contained higher titers of *A. actinomycetemcomitans*-specific IgG antibodies than the healthy control sera) increased ROS production compared to the results of opsonization with healthy control sera. Human IgG antibodies are needed for the efficient opsonization and phagocytosis of *A. actinomycetemcomitans* by neutrophils [77]. Moreover, active complement is needed together with *A. actinomycetemcomitans*-specific IgG antibodies for the enhancement of opsonization and subsequent killing of bacteria by neutrophils [77], whereas complement alone is not an effective opsonizing agent [78]. These *A. actinomycetemcomitans-*specific IgG antibodies that may enhance phagocytosis are mainly directed against LPS, fimbriae, an OmpA-like 29-kDa protein, RcpA and C, TadA, YaeT, and TufA [79–84]. Among the known neutrophil receptors, TLR2, which recognizes gram-negative outer membrane lipoproteins, is needed for the efficient phagocytosis of *A. actinomycetemcomitans* cells by mouse neutrophils [85,86]. In addition, platelets augment the rate of phagocytosis by improving the binding of *A. actinomycetemcomitans* to neutrophils and enhancing neutrophil activation in a process involving TLR2-mediated platelet-neutrophil aggregation [86]. In addition to minimal ROS production, the recombinant 165-amino acid-long BilRI polypeptide did not induce IL-6 production by THP-1 macrophages. The results indicate only that the BilRI polypeptide cannot stimulate macrophages, since recombinant BilRI did not contain the lipid component that is attached to the N-terminal cysteine of the native form of protein [29]. It is most likely that native mature cell-free BilRI can stimulate macrophages by binding to a TLR2-containing receptor homodimer with its N-terminal lipid component, similar to what is observed for other gram-negative lipoproteins [87]. To study this topic in greater detail, large amounts of native BilRI need to be purified from *A. actinomycetemcomitans*, which may be a difficult task considering the relatively low expression levels of BilRI.

The deletion of the *bilRI* gene did not have any effect on the antimicrobial susceptibility of *A. actinomycetemcomitans*. Various OMPs, consisting mostly of porins or other pore-forming proteins of gram-negative species, have been shown to be involved in antimicrobial susceptibility/resistance [88,89]. In addition to participating in systems involving efflux pumps, OMPs may affect the composition of the extracellular matrix, which can protect cells from antimicrobial agents. In particular, eDNA is known to bind and thereby decrease bacterial susceptibility to β-lactams [90,91]. Our earlier study suggested that the OM secretin HofQ provides some protection against β-lactams in *A. actinomycetemcomitans* [36]. The Δ*hofQ* mutant shows lower amounts of both eDNA and proteins in its biofilm matrix than the wild-type parental strain [36], whereas the Δ*bilRI* mutant shows slightly lower amounts of eDNA and higher amounts of proteins than the wild-type strain [30]. In conclusion, it was found that physiological amounts of BilRI in the OM of *A. actinomycetemcomitans* could not mediate resistance to any of the tested antibiotics.

It has been suggested that during anhydrobiosis outside of their hosts, nematodes and larvae of species such as *Steinernema carpocapsae, Aphelenchus avenae*, and *Caenorhabditis elegans* express various LEA proteins, which are highly hydrophilic and intrinsically disordered [92–94]. These LEA proteins might protect the parasitic nematodes from environmental stress while it is trying to find a new host. Humans and some Old World primates are the only natural hosts of *A. actinomycetemcomitans* [95–97], and person-to-person transmission (both vertical (from parent to child) and horizontal (from spouse to spouse)) has been reported in families [98,99]. It is likely that the species encounters stressful conditions, such as low ambient temperature, outside of the host. The LEA-like protein BilRI might protect the pathogen in these conditions and potentially increase the transmission potential. However, the significance of the possible role of BilRI in the transient life cycle outside the host needs further study.

## Supplementary Material

Supplemental Material

## Data Availability

The data of BilRI chemical shifts that support the findings of this study are openly available in BMRB database at www.bmrb.wisc.edu, reference number 27,824.
