# Supplementary material for: Decreased temperature increases the expression of a disordered bacterial late embryogenesis abundant (LEA) protein that enhances natural transformation
Source: Virulence. 2021 May 3;12(1):1239–57. doi: 10.1080/21505594.2021.1918497 (PMC8096337; doi:10.1080/21505594.2021.1918497)
Supplement: Supplemental Material [file KVIR_A_1918497_SM0391.zip › Final_Supplemental_material.docx]

**Supplemental Materials and Methods**

**Investigation of the natural transformation efficiency**

The effect of *bilRI*-deletion mutation on the efficiency of DNA-uptake from the surrounding environment by *A. actinomycetemcomitans* strains D7S (wildtype), the markerless *bilRI*-deletion mutation strain (Δ*bilRI*) [1], and a mutant strain in which spectinomycin resistance cassette had been substituted for *bilRI* (Δ*bilRI*::*spe*^r^, *i.e.*, the intermediate stage while creating the markerless Δ*bilRI*-mutant [1]) was examined by performing natural transformation using linear DNA-stretches that comprised of an antibiotic resistance cassette flanked by stretches of DNA sequence complementary to the genomic DNA of *A. actinomycetemcomitans*. The flanking stretches contained sequences complementary to the sequences upstream and downstream of *bilRI*. In between of these stretches was cloned either spectinomycin resistance cassette (to be used for natural transformation of the markerless Δ*bilRI*-strain), or kanamycin resistance cassette (for Δ*bilRI*::*spe*^r^-strain). For the *spe^r^*-linear construct the *bilRI* upstream and downstream sequences were amplified from genomic *A. actinomycetemcomitans* D7S DNA using primers that produced suitable restriction sites to the PCR-products (sixA_FD_SalI and phoGlu_R_SmaI for the upstream stretch; ycgL_FD_SacI and bilRI_RD_BamHI for the downstream stretch). The products were then digested with the corresponding restriction enzymes and ligated with *spe^r^*-cassette (digested from the plasmid pLox2-W-spe (a generous gift from Prof. Casey Chen, USC, LA, CA, USA) with SalI and BamHI and pUC19-vector base (digested with SmaI and SacI). For the *kan^r^*-linear construct the *bilRI*-downstream sequence was amplified from genomic DNA as described above, whereas the *bilRI*-upstream sequence was a synthetic DNA-stretch flanked by XbaI and PstI restriction sites (pEX-a128-Fr_BbilRI_US_short from Eurofins Genomics). These upstream and downstream sequences were ligated with *kan^r^*-cassette [digested from pVT1503 (a generous gift from Dr. Jan Oscarsson, Umeå University, Umeå, Sweden) with PstI and after isolation of the resultant *kan^r^*-fragment from agarose gel further with BamHI] and pUC19 vector base (digested with SacI and XbaI). The newly formed vector plasmids were then transformed into *E. coli* XL-1 Blue (Stratagene) using electroporation. Successful transformants were selected based on their ability to grow on LA-plates supplemented with both ampicillin (resistance mediated by pUC19) and either spectinomycin or kanamycin (resistance mediated by the corresponding cassettes included in the cloned sequences). The vector plasmids were then amplified in the XL-1 Blue strain followed by isolation and purification using GeneJet MiniPrep Purification-kit (ThermoScientific). The plasmids were then digested with suitable restriction enzymes (*bilRI*-upstream_spe^r^_*bilRI*-downstream: SmaI and SacI; *bilRI*-upstream_kan^r^_*bilRI*-downstream: SacI and XbaI) to produce the final linear DNA-stretches, in which the 5´overhangs were filled using the Klenow fragment (ThermoFisher Scientific).

The blunt-end DNA-stretches were used for natural transformation, performed by following the method described by [2], with some modifications. The different *A. actinomycetemcomitans* strains were grown on horse serum-supplemented TSA-plates for 3-4 days in a candle jar at 37°C, after which they were collected in mTSB and the turbidity of the suspension measured at 600 nm. Of each strain two droplets, each containing 2x10^7^ cells, were then spotted on a horse serum-supplemented TSA-plate by carefully pipetting to avoid a runoff of the droplets. The droplets were then dried, allowing the spotted cells to attach to the plate, by growing the plates (with lids up) in a candle jar at 37°C for 2 h. The cells were then incubated with the linear DNA constructs (25 ng DNA per spot) by adding the DNA on top of the cells in a small volume (<10 µL) and carefully mixing with a metal wire loop. The plates were then grown in a candle jar at 37°C for 5 h to allow the cells to take up the DNA. Cells from the two spots were then collected into a 100 µL suspension of mTSB and plated on horse serum-supplemented TSA-plates that contained either 50 µg/mL spectinomycin or 30 µg/mL kanamycin, depending on the linear construct. The plates were grown in candle jars at 37°C for at least 3 days, after which the number of transformant colonies was calculated on each plate.

**Cloning of *dsred* gene and expression of RFP under genomic *bilRI* promoter in *A. actinomycetemcomitans***

To study the activity of the promoter region of the *bilRI* gene, the gene encoding *bilRI* (NC_017846.1 AaD7S_02241) was replaced by the *dsred-M1* gene preserving the promoter region of *bilRI*. The *dsred-M1* gene was introduced to *A. actinomycetemcomitans* using natural transformation of a linear DNA construct that contained the sequence for the *dsred-M1* gene followed by the sequence for the spectinomycin resistance cassette. At both ends of the construct were inserted sequences that flanked the *bilRI* gene in both upstream and downstream directions.

Synthetic DNA for *dsred-M1*, flanked by 5´-terminal KpnI and MfeI, and 3´-terminal SalI restriction sites (*dsred^A.a^*) with optimized codon usage for *E. coli* expression (Table 1), was ordered from Eurofins Genomics. The *spe* cassette, flanked by SalI and BamHI restriction sites as well as *loxP* sites, was from a pLox2-Spe plasmid [3] which was a generous gift from Professor Casey Chen (University of Southern California, Los Angeles, CA, USA). The *dsred^A.a^* gene and the *spe* cassette were ligated into the pUC19 vector [4] and the pUC19_dsred^A.a^_spe plasmid then transformed into the XL1 Blue *E. coli* competent strain (Stratagene). The transformants were screened on LA-plates supplemented with 100 μg/mL ampicillin and 50 μg/mL spectinomycin.

To amplify the upstream and the downstream regions of the *bilRI* gene, two PCR fragments were generated outgoing from a 2960-bp PCR product that contained the *bilRI* gene and that was amplified from genomic DNA of *A. actinomycetemcomitans* D7S using bilRI_nest primers and Phusion High-Fidelity DNA Polymerase (Thermo Fisher Scientific). The sequences of all primers are listed in Table S1: The upstream region was amplified with the primer pair pbilRI_FD_XhoI/phoGlu_R and the downstream region with the primer pair ycgL-FD/bilRI_RD_BamHI. The PCR fragments were then digested with MfeI (restriction site appeared in the *bilRI* promoter region) or BamHI restriction enzymes. The pUC19_dsred^A.a^_spe plasmid was digested with MfeI and BamHI. The digested PCR fragments and the digested *dsred_spe* fragment were then ligated with T4 DNA ligase for 4 h in ambient temperature. The ligation was inactivated at 65°C for 10 min and the linear *bilRI_upstream__dsred^A.a^_spe_bilRI_downstream_* construct was then used in natural transformation of the D7S.

Natural transformation was performed according to a previously described method [2] with some modifications. In brief, *A. actinomycetemcomitans* strain D7S was recovered from -80°C storage cultures on modified tryptone soy agar plates (3 % tryptone soy broth (TSB; Lab-M, Lancashire, UK), 0.3 % yeast extract (Lab-M), 1.5 % agar, 5 % heat-inactivated horse serum (HyClone, SH30074.03, Thermo Fisher Scientific)) at 37°C in a candle jar for 3 days. A suspension of cells was then prepared in 3 % TSB supplemented with 0.6 % yeast extract, and grown overnight. The bacterial cell number in the suspension was estimated according to the method described by Karched et al. [5]. 2 x 10^7^ colony forming units (CFUs) were spotted on TSA-plates in a small area and grown at 37⁰C in a candle jar for 2 h. The cells were then mixed with the *bilRI_upstream__dsred^A.a^_spe_bilRI_downstream_* construct (~500 ng DNA supplemented with 1 mM CaCl_2_ per cell spot) using a metal wire loop and grown at 37⁰C in a candle jar for 5 h. The cells were then scraped up, resuspended in a small volume of TSB and plated on TSA-plates supplemented with 50 μg/mL spectinomycin. Colony PCR was used to confirm the deletion of the *bilRI* gene and the presence of the *dsred^A.a^_spe* construct in the genome of the transformed *A. actinomycetemcomitans*. Briefly, a loopful of bacteria was suspended in lysis buffer (20 μg/mL proteinase K and 2.5 % glycerol in 10 mM Tris-HCl, pH 7.5) and lysed at 50 ⁰C for 1 h and 95 ⁰C for 1 min. 20 μL of the lysis suspension was then added to a PCR reaction using bilRI_nest or OM_F_bilRI1/OM_R_bilR1 primers together with Taq DNA Polymerase (Thermo Fisher Scientific). The PCR products were then analyzed on an agarose gel. Mutant strains that contained the correct ~4.3-kbp product of the bilRI_nest primers (denoting insertion of the *dsred^A.a^_spe* fragment) and lacked the ~560-bp product of the OM_bilRI primers (denoting deletion of the *bilRI* gene) were selected for further studies.

To avoid potential distraction of the transcription of *dsred* by the high transcription levels of the *spe* cassette when grown on selective media, the *spe* cassette was removed from the ΔbilRI::dsred^A.a^_spe strains using the site-specific Cre/*loxP* gene deletion system as previously described [3, 6]. In brief, electrocompetent primary mutants were prepared and transformed with the pAT-Cre plasmid that contained the genes for *cre* recombinase and tetracycline resistance. The pAT-Cre plasmid was a generous gift from Professor Casey Chen (University of Southern California, Los Angeles, CA, USA). The cells were incubated with the pAT-Cre plasmid on ice for 1 h followed by electroporation (1250 V, 5 ms) and recovery in TSB medium (supplemented with 5 % heat inactivated horse serum) at 37°C in a candle jar for 2 h. The transformants were screened on TSA-plates supplemented with 6 μg/mL tetracycline. After 5 days of growth each visible colony was further plated on a TSA plate with no antibiotic, on a plate with 6 μg/mL tetracycline and on a plate with 50 μg/mL spectinomycin. Colonies sensitive for both antibiotics were considered potential markerless *ΔbilRI::dsred^A.a^* mutants. The deletion of the *bilRI* gene, the presence of the *dsred* gene and the removal of the *spe* cassette in these mutants were verified by colony PCR as described above, with the additional use of the Spe_UPrf/Spe_DWR2 primer pair for detection of the *spe* cassette.

**Table S1.** Primer sequences that were used in producing *A. actinomycetemcomitans* strain which produced RFP under the genomic *bilRI* promoter.

| Primer name | Sequence | Annealing T_m_ (°C) |
| --- | --- | --- |
| bilRI_nest-F | 5´-GTATGGTGCCTGACTTTCGG-3´ | 62.5 Phusion/52.2 Taq |
| bilRI_nest-R | 5´-TTATGGTGGATCACCTTGGT-3´ | 62.5 Phusion/52.2 Taq |
| pbilRI_FD_XhoI | 5´-ATACTCGAGCGTTACTCCTAAATTAACAATTG-3´ | 56 Phusion |
| phoGlu_R | 5´-GCGACCAAGCCTTATTTA-3´ | 56 Phusion |
| ycgL_FD | 5´-CCAAGGCTGGAAAGCGATATT-3´ | 61 Phusion |
| bilRI_RD_BamHI | 5´-CTAGGATCCTGAAAGCAAATAAAAAAGCAGTCTA-3´ | 61 Phusion |
| OM_F_bilRI1 | 5´-ATACATATGAAAAAATCAGTATTAGCC-3´ | 50.2 Taq |
| OM_R_bilR1 | 5´-ATACTCGAGTTATTTGCTTTCAGTTTC-3´ | 50.2 Taq |
| Spe_UPrf | 5´-TGCAGGTCGATTTTCGTTCGT-3´ | 56.7 Taq |
| Spe_DWR2 | 5´-GCCACTGCATTTCCCGCATA-3´ | 56.7 Taq |

**Supplemental References**

[1] Ahlstrand T, Tuominen H, Beklen A, Torittu A, Oscarsson J, Sormunen R, Pöllänen MT, Permi P, Ihalin R. A novel intrinsically disordered outer membrane lipoprotein of Aggregatibacter actinomycetemcomitans binds various cytokines and plays a role in biofilm response to interleukin-1beta and interleukin-8. Virulence 2017; 8:115-34; PMID: 27459270; DOI: 10.1080/21505594.2016.1216294 [doi].

[2] Wang Y, Goodman SD, Redfield RJ, Chen C. Natural transformation and DNA uptake signal sequences in Actinobacillus actinomycetemcomitans. J Bacteriol 2002; 184:3442-9; PMID: 12057937.

[3] Cheng YA, Jee J, Hsu G, Huang Y, Chen C, Lin CP. A markerless protocol for genetic analysis of Aggregatibacter actinomycetemcomitans. J Formos Med Assoc 2014; 113:114-23; PMID: 24530245; DOI: 10.1016/j.jfma.2012.05.005 [doi].

[4] Yanisch-Perron C, Vieira J, Messing J. Improved M13 phage cloning vectors and host strains: Nucleotide sequences of the M13mp18 and pUC19 vectors. Gene 1985; 33:103-19; PMID: 2985470; DOI: 0378-1119(85)90120-9 [pii].

[5] Karched M, Paul-Satyaseela M, Asikainen S. A simple viability-maintaining method produces homogenic cell suspensions of autoaggregating wild-type Actinobacillus actinomycetemcomitans. J Microbiol Methods 2007; 68:46-51; PMID: 16904783; DOI: S0167-7012(06)00190-4 [pii]; 10.1016/j.mimet.2006.06.004 [doi].

[6] Fujise O, Wang Y, Chen W, Chen C. Adherence of Aggregatibacter actinomycetemcomitans via serotype-specific polysaccharide antigens in lipopolysaccharides. Oral Microbiol Immunol 2008; 23:226-33; PMID: 18402609; DOI: 10.1111/j.1399-302X.2007.00416.x [doi].

**Figure texts**

**Figure S1.** BilRI is conserved at the amino acid level in *A. actinomycetemcomitans* strains. In most cases, the amino acid differences did not change the biophysical characteristics of the side chain.

**Figure S2.** *A. actinomycetemcomitans* BilRI sequences were divided into roughly five distinct clusters in a dendrogram generated according to their amino acid sequences. The RhAA1 strain was isolated from rhesus macaques, and the rest of the strains were isolated from human hosts.

**Figure S3.** *A. actinomycetemcomitans* D7S BilRI shared sequence similarity with various plant LEA proteins. However, the BilRI sequence was significantly shorter than those of the plant LEA proteins.

**Figure S4**. Section of an overlay of the ^1^H, ^15^N HSQC spectra of free BilRI (red) and BilRI in the presence of LPS (approx. 2 mg/ml) (blue). Residues affected by LPS are labeled in the spectra and highlighted in the BilRI amino acid sequence below. Perturbations in several mutually distant stretches of the BilRI sequence could be explained by either the folding of BilRI or simultaneous interaction with more than one LPS molecule.
