# Supplementary figures and images for: Decreased temperature increases the expression of a disordered bacterial late embryogenesis abundant (LEA) protein that enhances natural transformation
Source: Virulence. 2021 May 3;12(1):1239–57. doi: 10.1080/21505594.2021.1918497 (PMC8096337; doi:10.1080/21505594.2021.1918497)

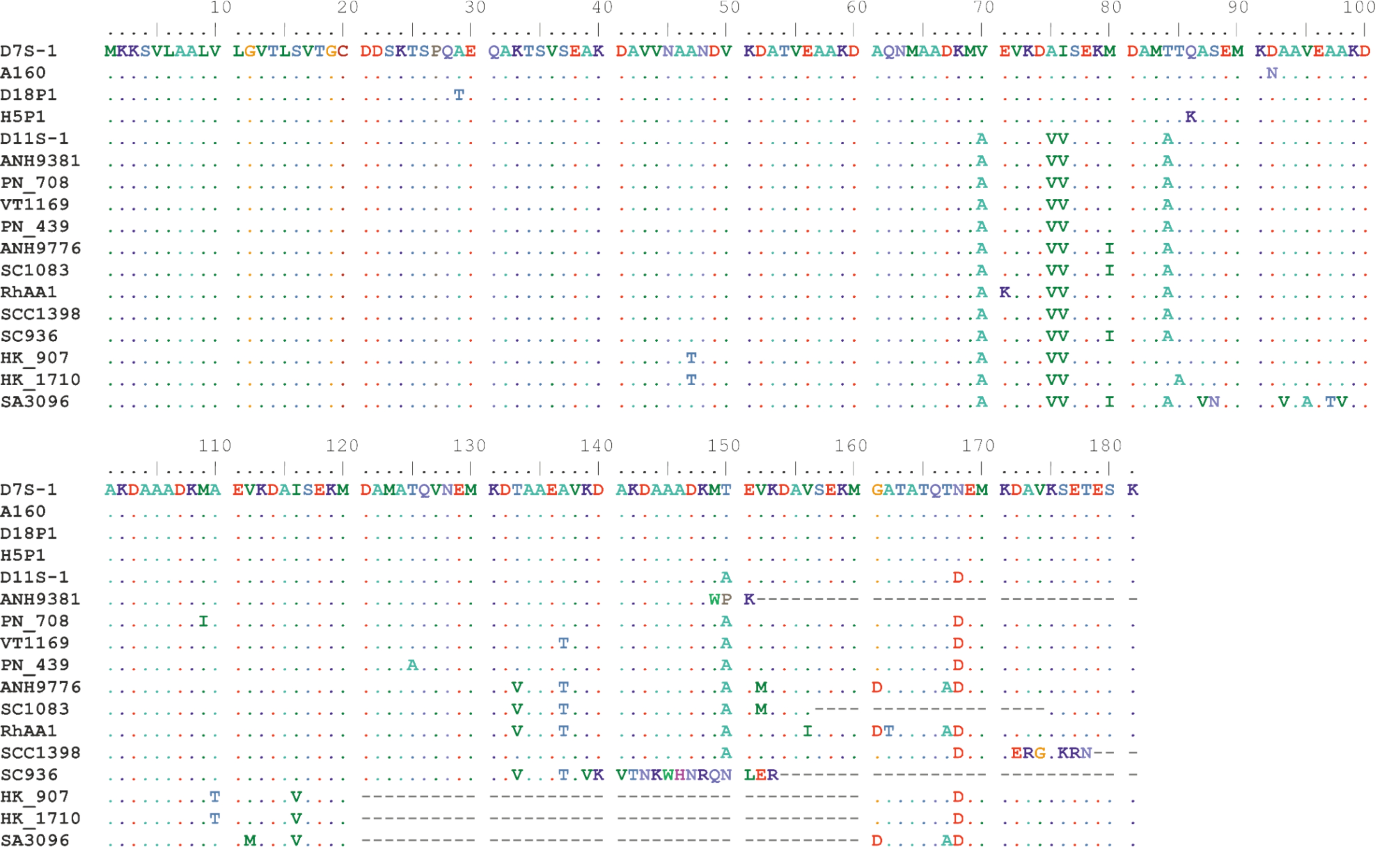

Supplement: Supplemental Material [file KVIR_A_1918497_SM0391.zip › Figure_S1.jpg]

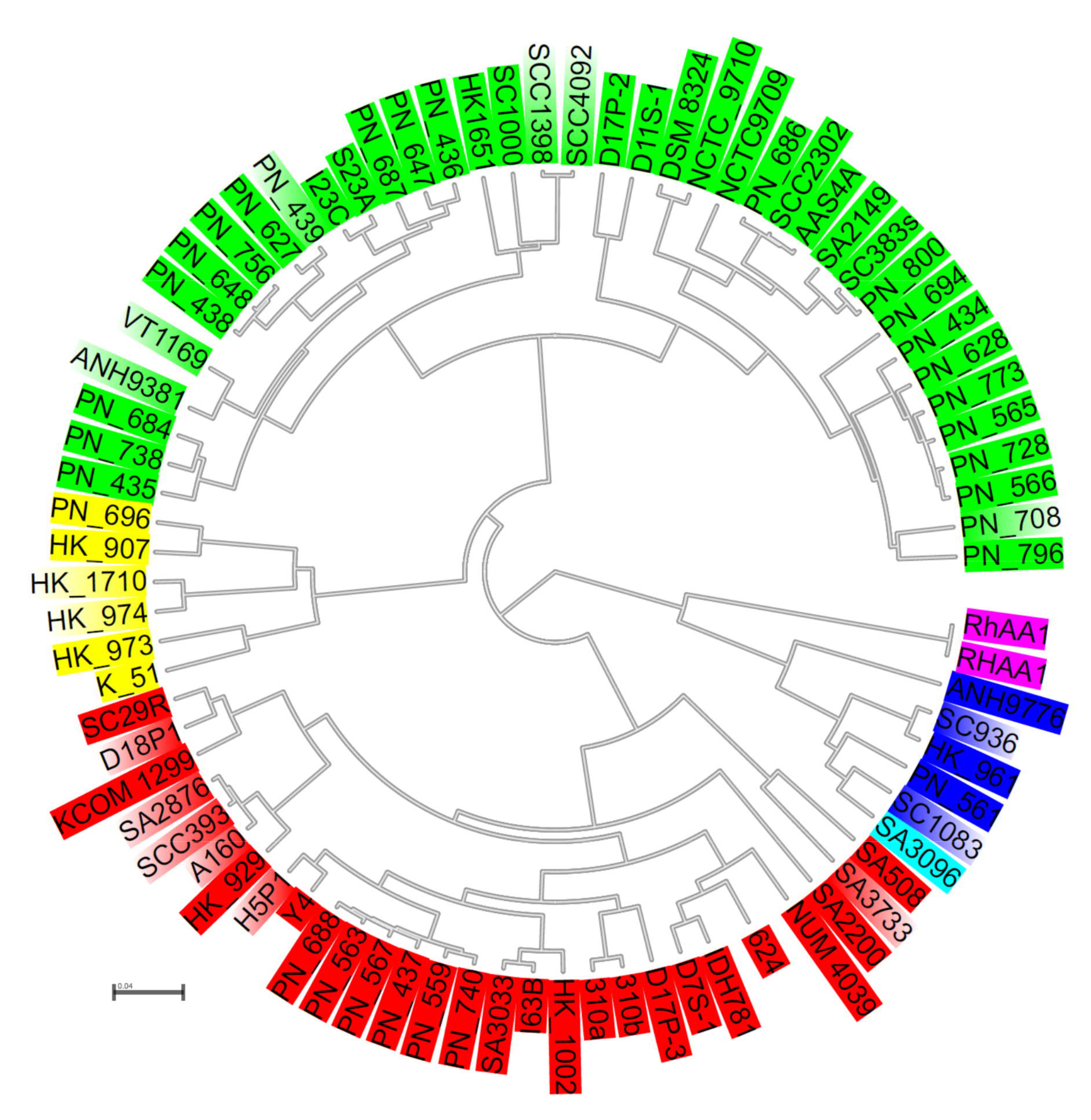

Supplement: Supplemental Material [file KVIR_A_1918497_SM0391.zip › Figure_S2.jpg]

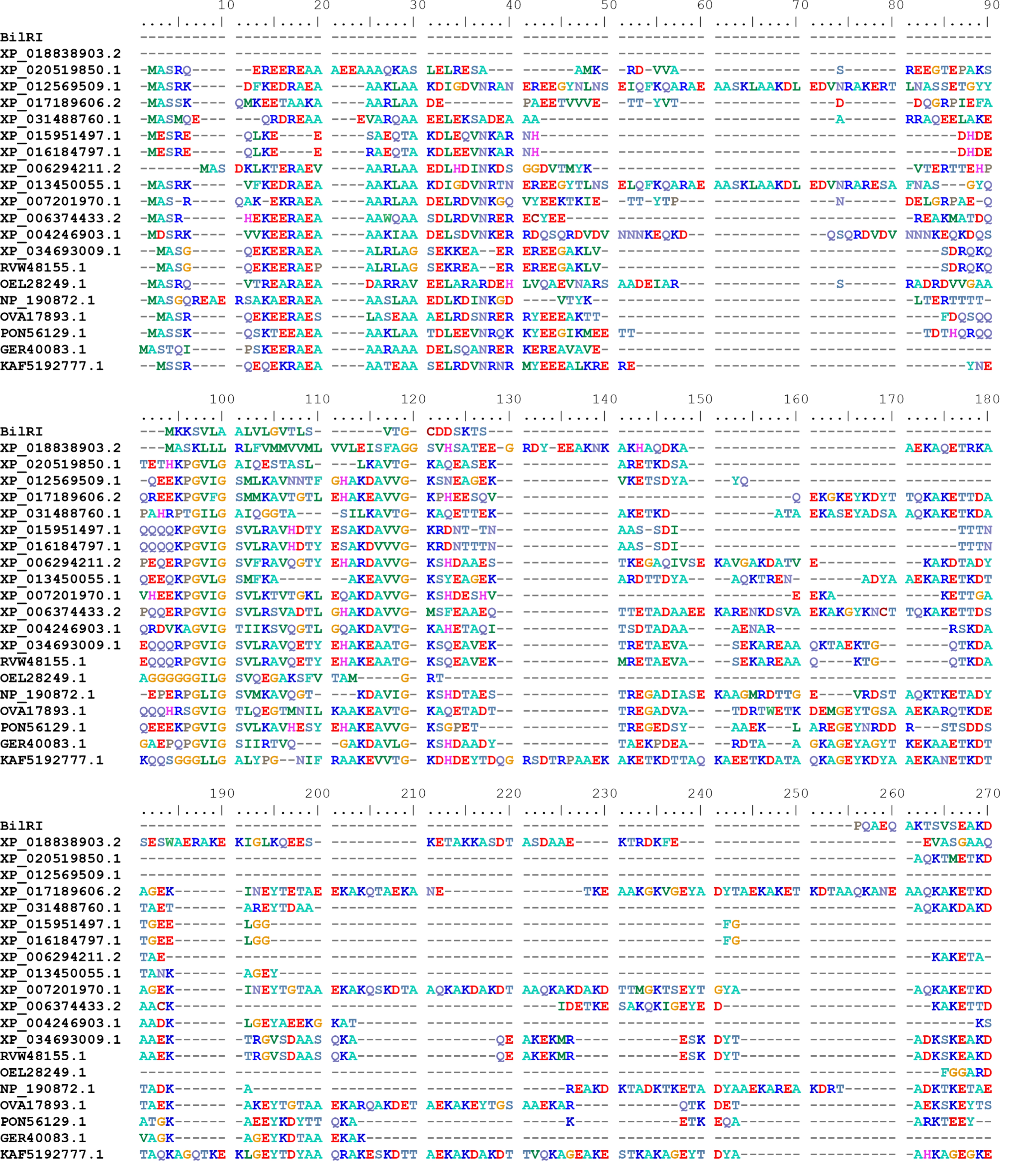

Supplement: Supplemental Material [file KVIR_A_1918497_SM0391.zip › Figure_S3.1.jpg]

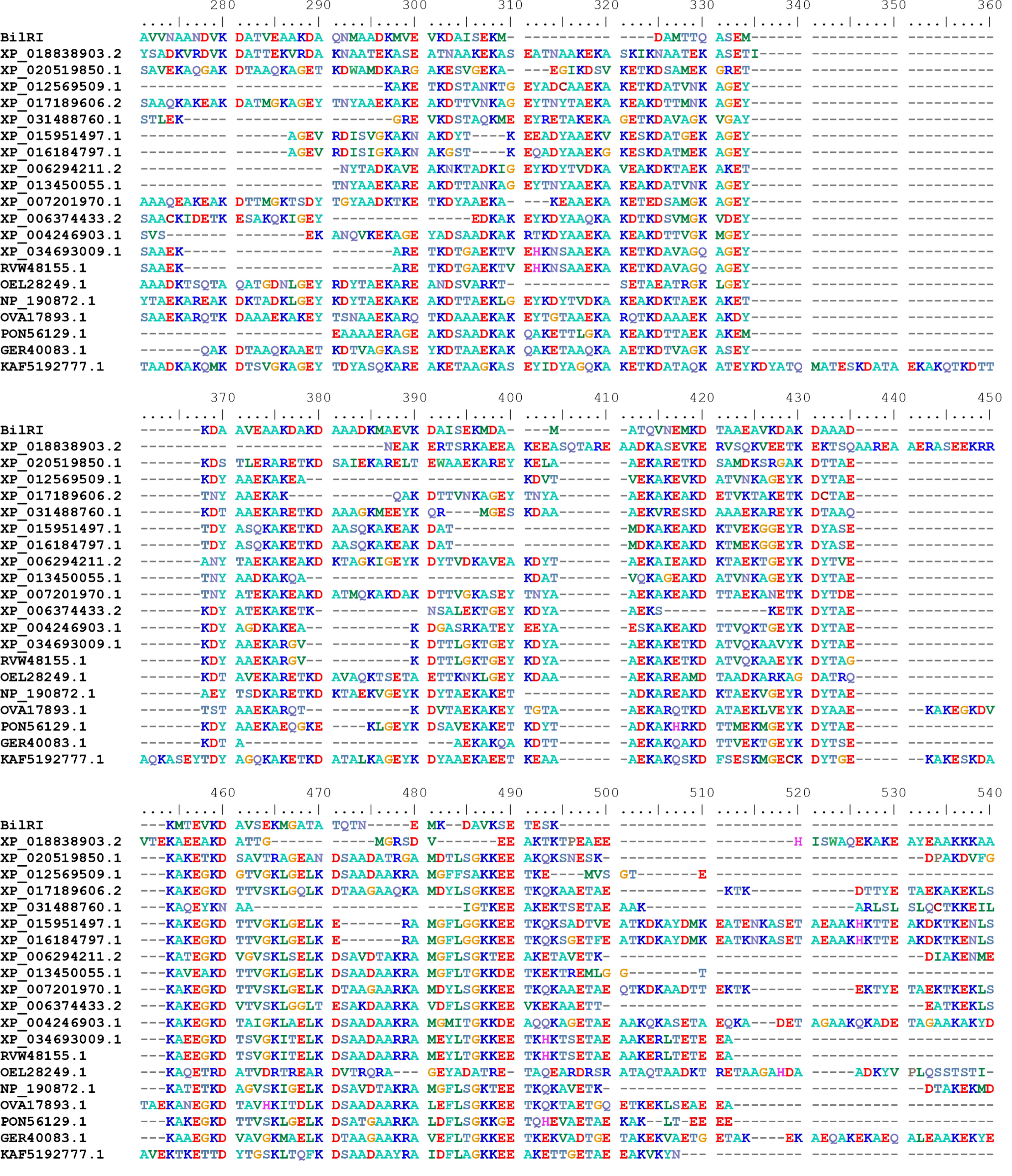

Supplement: Supplemental Material [file KVIR_A_1918497_SM0391.zip › Figure_S3.2.jpg]

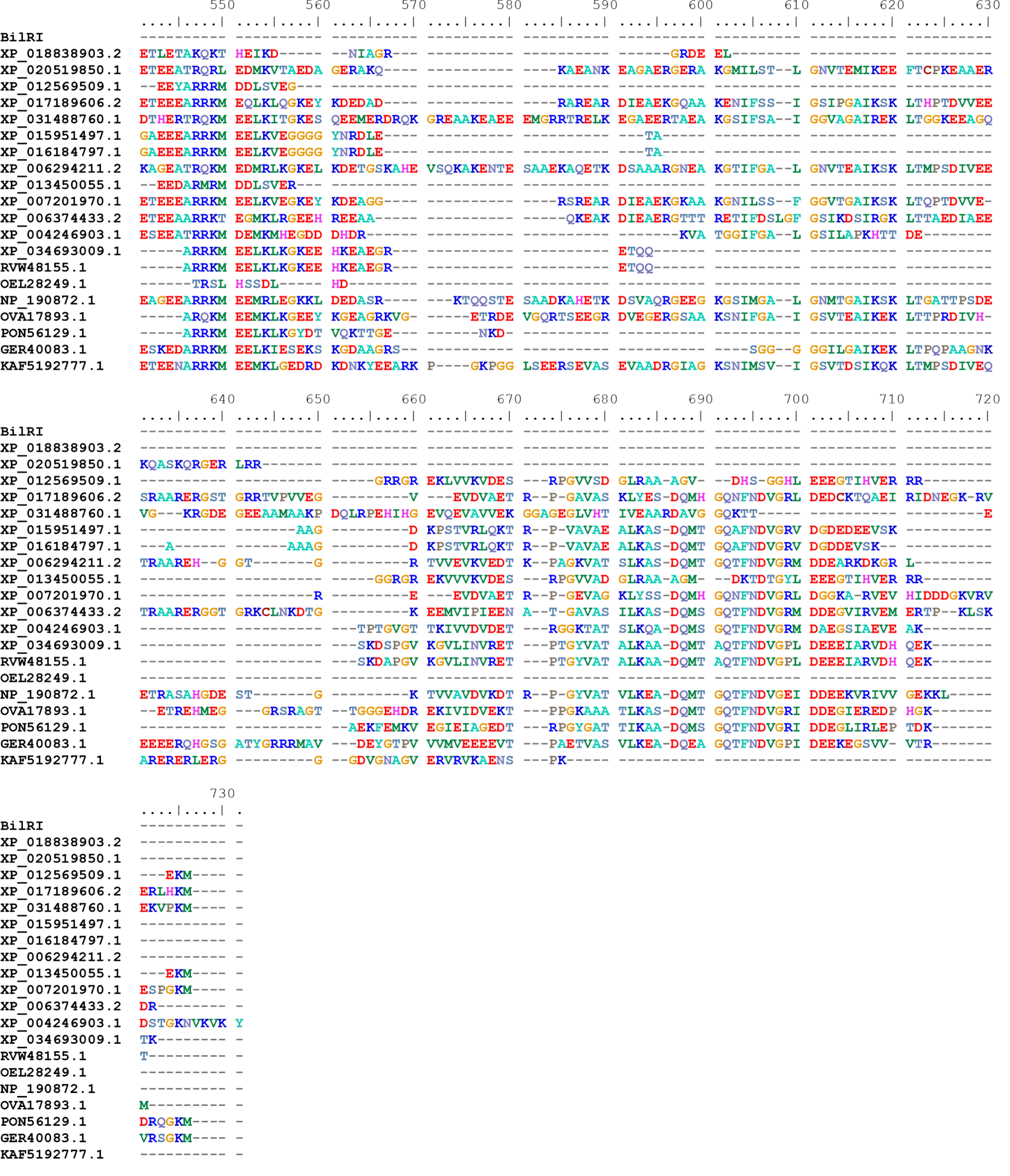

Supplement: Supplemental Material [file KVIR_A_1918497_SM0391.zip › Figure_S3.3.jpg]

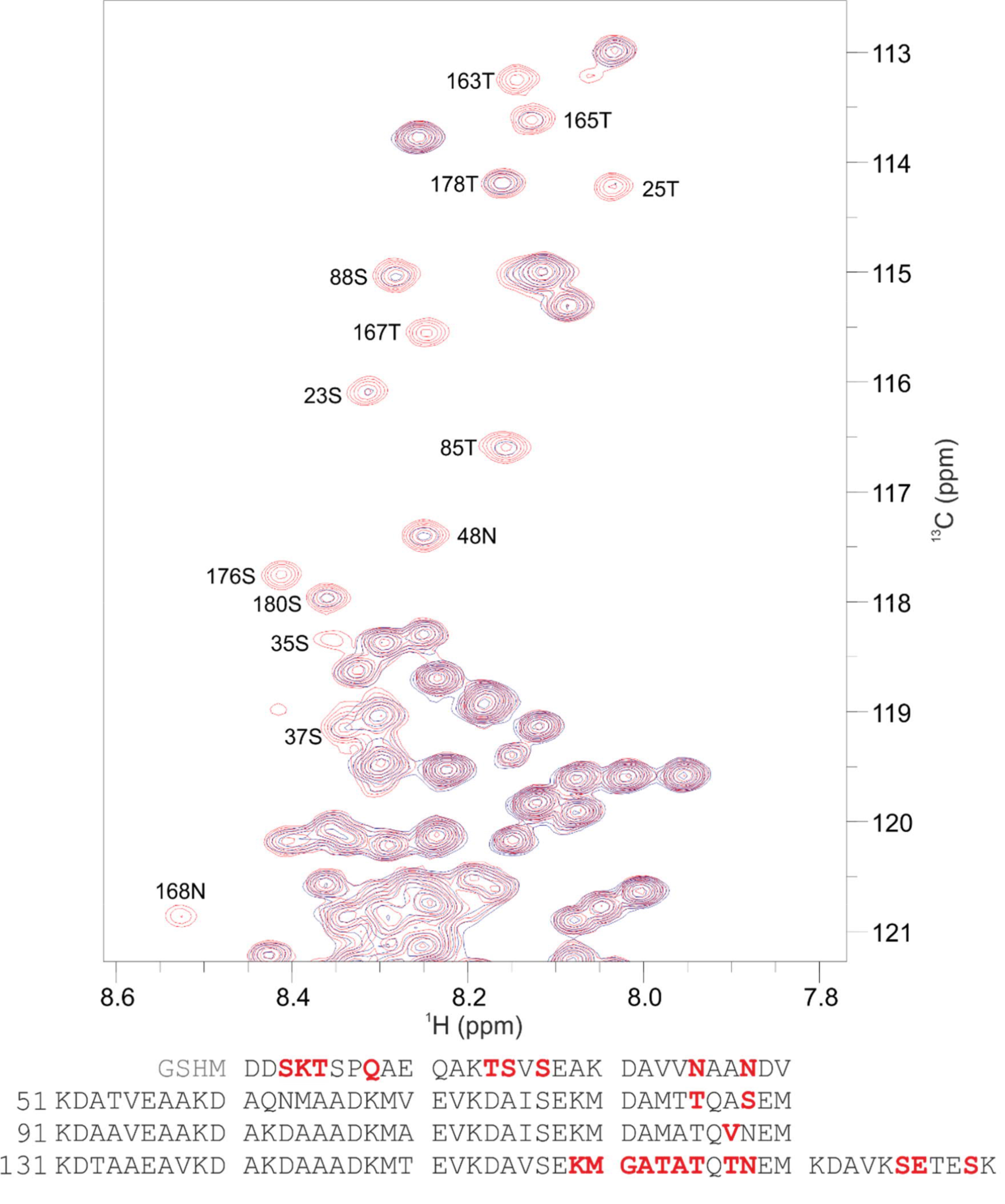

Supplement: Supplemental Material [file KVIR_A_1918497_SM0391.zip › Figure_S4.jpg]
